# Utility of Artificial Intelligence Plaque Quantification: Results of the DECODE Study

**DOI:** 10.1016/j.jscai.2024.101296

**Published:** 2024-03-26

**Authors:** Sarah Rinehart, Steven J. Raible, Nicholas Ng, Sarah Mullen, Whitney Huey, Campbell Rogers, Amit Pursnani

**Affiliations:** a Charleston Area Medical Center (CAMC), Charleston, West Virginia; b Norton Healthcare, Louisville, Kentucky; c HeartFlow, Mountain View, California; d Department of Medicine, Division of Cardiology, NorthShore University Health System, Evanston, Illinois

**Keywords:** artificial intelligence quantified coronary plaque analysis, atherosclerosis, coronary artery disease, coronary computed tomographic angiography, coronary plaque, coronary plaque quantification

## Abstract

**Background:**

Artificial Intelligence Plaque Analysis (AI-QCPA, HeartFlow) provides, from a CCTA, quantitative plaque burden information including total plaque and plaque subtype volumes. We sought to evaluate the clinical utility of AI-QCPA in clinical decision making.

**Methods:**

One hundred cases were reviewed by 3 highly experienced practicing cardiologists who are SCCT level 3 CCTA readers. Patients had varying levels of calcium (median CACS: 99.5) and CAD-RADS scores. Initial management plan for each case was a majority decision based upon patient demographics, clinical history, and CCTA report. AI-QCPA was then provided for each patient, and the plan was reconsidered. The primary endpoint was the reclassification rate (RR). In a secondary analysis of 40 cases, the above process was repeated but the initial plan was based upon review of the actual CCTA images.

**Results:**

RR following AI-QCPA review was 66% (66/100) of cases (95% CI, 56.72%-75.28%). RR ranged from 47% in cases with CACS 0 to 96% in cases with CACS >400, and from 40% in CAD-RADS 1 cases to 94% in CAD-RADS 4 cases. RR was higher in cases with coronary stenoses ≥50% (89.5%) vs cases with stenoses <50% (51.6%). RR was 39% in cases with LDL <70 mg/dL vs 70% in LDL ≥70 mg/dL. Following review of the CCTA images rather than the CCTA report, the RR was 50% (95% CI of 34.51% - 65.49%). The primary reclassification effect was to intensify preventative medical therapy.

**Conclusions:**

Adding AI-QCPA to CCTA alone leads to a change in clinical care in two-thirds of patients.

## Introduction

Quantifying and characterizing coronary atherosclerotic plaque as a component of coronary computed tomographic angiography (CCTA) reporting may provide additional information useful for clinical decision-making. To date, plaques have been quantified manually by CCTA readers which is time-consuming and less reproducible than using an artificial intelligence (AI)-based process.^1^

Artificial intelligence quantified coronary plaque analysis (AI-QCPA) provides quantitative plaque information calculated from a standard CCTA and volumetric quantification for total plaque and plaque subtypes. Recent clinical studies, REVEALPLAQUE^2^ and MIAMI^3^ have shown excellent agreement of HeartFlow's AI-QCPA with invasive intravascular ultrasound (IVUS) quantification of coronary plaque. Furthermore, studies have shown that plaque parameters derived from HeartFlow’s AI-QCPA inform patient-specific risk of cardiac events.^4^

Although coronary plaque characterization and quantification via CCTA is possible, its utility in terms of impact on patient management remains unclear. The DEcisions for Treating COronary Disease are Changed in Patients Evaluated With Quantified Plaque Analysis (DECODE) study aimed to determine how the incremental information of HeartFlow's AI-QCPA would change clinical decision-making and patient management.

## Methods

### Study design

**Figure 1:**
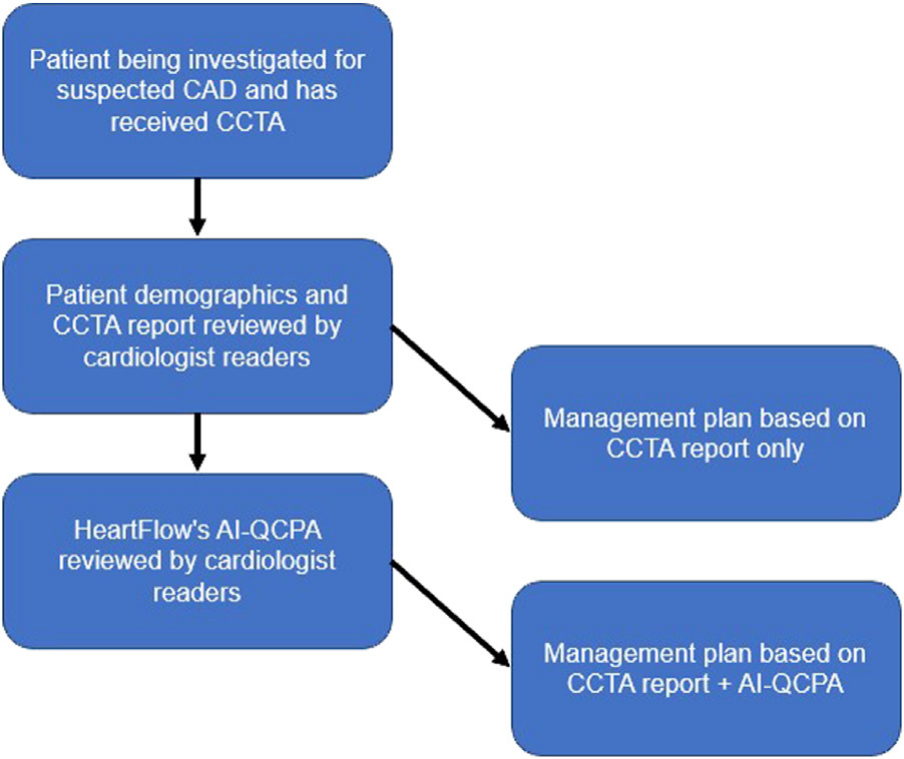
**Study design.** AI-QCPA, artificial intelligence quantified coronary plaque analysis; CAD, coronary artery disease; CCTA, coronary computed tomographic angiography.

**Figure 2:**
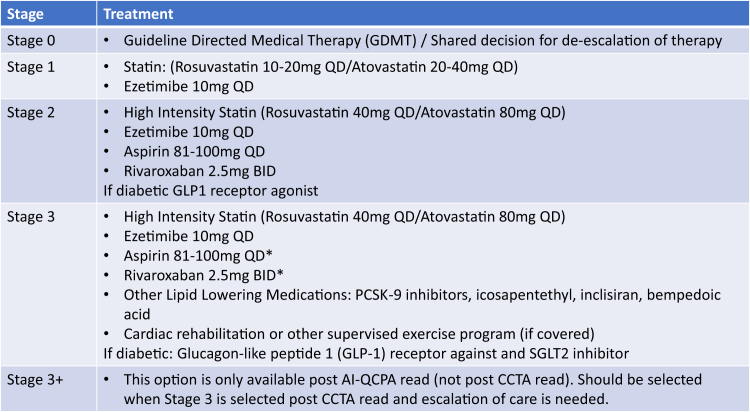
**Medical management staging**.^5^

This study involved the assessment of CCTA and AI-QCPA data for 100 patients. The population included patients with suspected coronary artery disease (CAD) who underwent a clinically indicated CCTA. In an in-person group setting, 3 experienced cardiologists, all of whom were Society of Cardiovascular Computed Tomography Level 3 CCTA readers (A.P., S.R., S.J.R.) were provided access to the CCTA report and to the patient’s age, sex, presenting symptoms, risk factors, smoking status, body mass index (BMI), creatinine, high-density lipoproteins, low-density lipoproteins (LDL), triglycerides, total cholesterol, and medications at the time of the CCTA. An initial management plan was then agreed upon by the majority as outlined below based on the CCTA report and clinical data (Figure 1). Possible management plans used a recently published hierarchy (Figure 2).^5^

**Figure 3:**
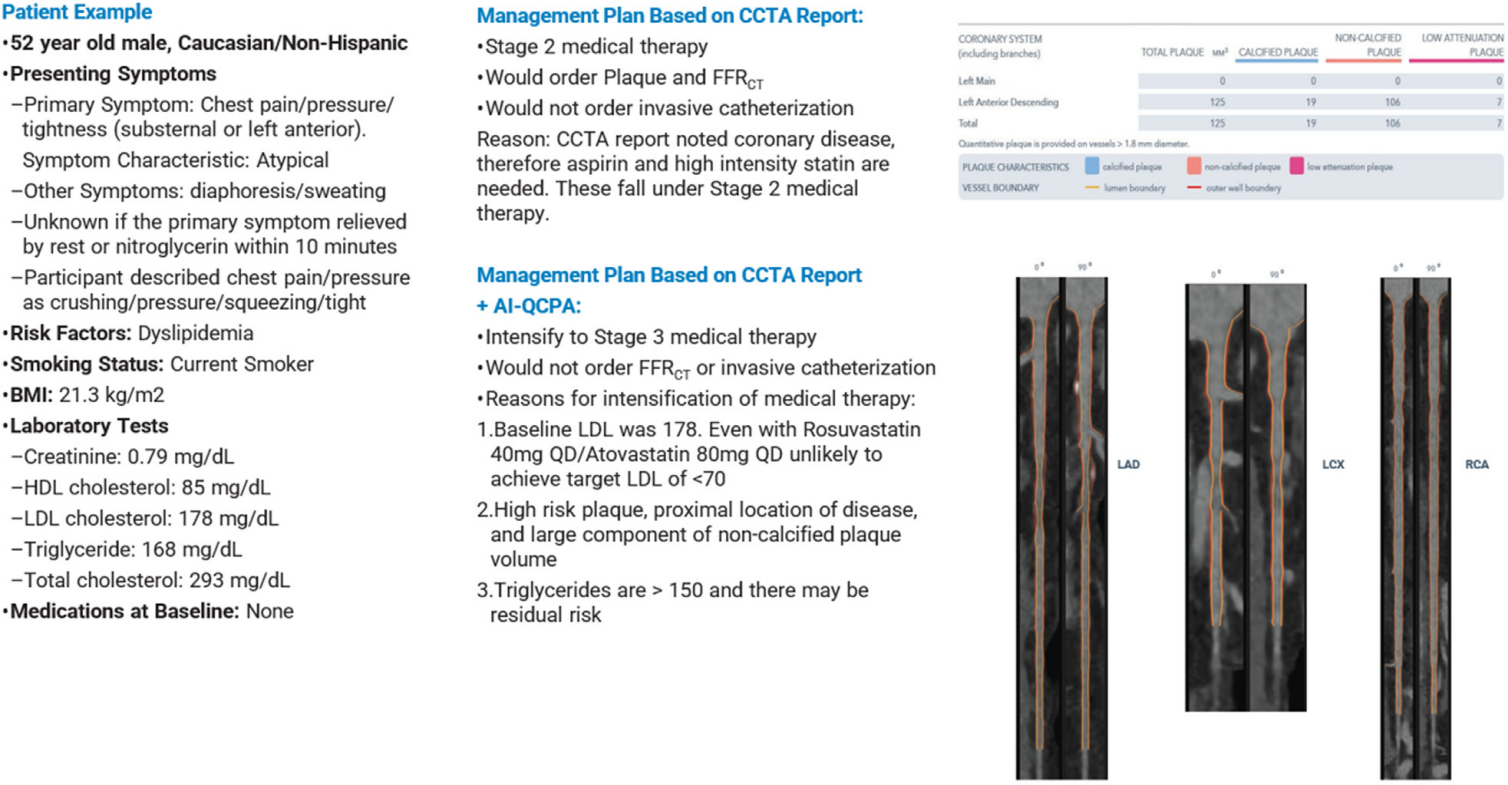
**Patient example.** AI-QCPA, artificial intelligence quantified coronary plaque analysis; CCTA, coronary computed tomographic angiography.

Once this decision was made for each case, HeartFlow's AI-QCPA data were revealed for that case, and the 3 cardiologists were again asked to decide, by majority, on a management plan choosing from the same options (Figure 1). A patient example is displayed in Figure 3.

The primary end point for this study was the difference between management plan made on the basis of interpretation of the CCTA alone compared to management plan incorporating AI-QCPA data.

### Patient selection

Patients were selected from the Prospective Randomized Trial of the Optimal Evaluation of Cardiac Symptoms and Revascularization (PRECISE) Trial^6^ data set spread between coronary artery calcium (CAC) buckets of 0, 1 to 99, 100 to 399, and 400+. As there were a limited number of patients in the 0 CAC bucket, all available 19 were included in the DECODE study. The remainder were randomly selected, 27 from each of the higher CAC buckets using a random number generator. All patients consented to have their data used in future research projects such as DECODE. The CCTAs were stratified by calcium score and CAD-RADS 2.0 score. The patients varied by age, sex, race, and disease burden, and CCTA scanners reflected all major manufacturers and a full range of image quality.

### AI-QCPA

AI-QCPA (HeartFlow) uses a patient-specific CCTA-derived 3D model of the arterial lumen and outer wall to quantify and characterize plaque. Cross-sectional analysis provides lumen, vessel, and plaque areas including calcified, noncalcified, and low-attenuation plaque areas. Total plaque volume (TPV) is calculated as the sum of volumes of noncalcified and calcified volumes.

### Exploratory analysis

In a secondary analysis of 40 CCTA cases, the above process was repeated but the initial management plan was based on review of the actual CCTA images with RadiAnt DICOM Viewer.

### Statistical analysis

A sample size of 100 was calculated to be the minimum number required to give a confidence level of 95% with a 0% margin of error and a response distribution of 50%. All continuous variables are expressed as mean ± SD. Categorical variables were presented as number (percent). For comparison of categorical variables (change in management plan after AI-QCPA), the percentage change and exact 95% CIs are presented. Statistical analyses were performed with the use of SAS software version 9.4 (SAS Institute).

## Results

### Patient demographic characteristics

The median patient age was 64 years, 41% were female, and median BMI was 30.1 kg/m^2^. Diabetes was present in 23%, dyslipidemia in 84%, hypertension in 71%, and family history of premature CAD in 48%. Median LDL concentration was 103 mg/dL and 42% were receiving statin treatment. Median coronary artery calcium score (CACS) was 99.5.

Management plan reclassification rate (RR) following AI-QCPA review was 66% (66/100) (95% CI, 56.72%-75.28%) (Central Illustration). RR ranged from 47% in cases with CACS 0% to 96% in cases with CACS >400 and from 40% in cases graded as CAD-RADS 1 to 94% in cases graded as CAD-RADS 4. RR was higher in cases with coronary stenoses ≥50% (89.5%) vs cases with stenoses <50% (51.6%). RR was 39% in cases with LDL <70 mg/dL vs 70% in cases with LDL ≥70 mg/dL (Table 1). RR of FFR_CT_ ordering was 24.2% in cases with <50% stenosis vs ≥50% stenosis (Table 2).

**Central Illustration:**
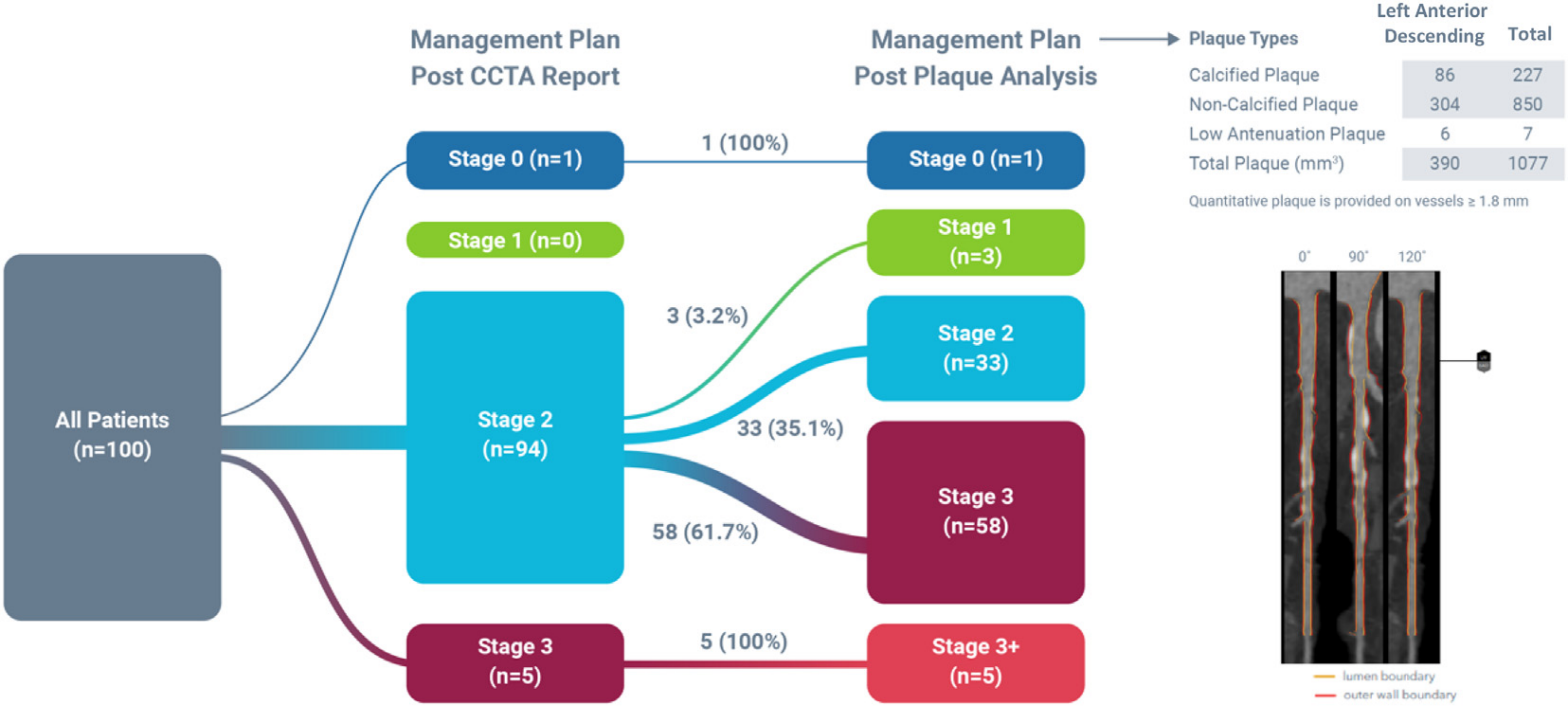
Reclassification rate following artificial intelligence quantified coronary plaque analysis. CCTA, coronary computed tomographic angiography.

**Table 1:** Primary end point: Reclassification of management plan from CCTA alone vs CCTA + AI-QCPA.

Cohort	Reclassification rate, n (%)
All cases	66 (66.0%) (95% CI, 56.72%-75.28%)
Calcium score 0	9 (47.4%)
Calcium score 1-99	14 (45.2%)
Calcium score 100-399	19 (76.0%)
Calcium score ≥ 400	24 (96.0%)
LDL < 70 mm/dL	5 (38.5%)
LDL ≥ 70 mm/dL	61 (70.1%)
LDL < 55 mm/dL	0 (0%)
LDL ≥ 55 mm/dL	66 (69.5%)
Stenosis ≥ 50%	34 (89.5%)
Stenosis < 50%	32 (51.6%)

AI-QCPA, artificial intelligence quantified coronary plaque analysis; CCTA, coronary computed tomographic angiography; LDL, low-density lipoprotein.

**Table 2:** Secondary end point: Reclassification of FFR_CT_ ordering in CCTA alone vs CCTA + AI-QCPA.

Cohort	Reclassification rate, n (%)
All cases	15 (15.0%)
Stenosis ≥ 50%	0 (0%)
Stenosis < 50%	15 (24.2%)

AI-QCPA, artificial intelligence quantified coronary plaque analysis; CCTA, coronary computed tomographic angiography.

In the exploratory analysis following the review of the CCTA images rather than the CCTA report, the RR was 50% (95% CI, 34.51%-65.49%) (Table 3).

**Table 3:** Exploratory end point: Reclassification management plan in CCTA images vs CCTA + AI-QCPA.

Cohort	Reclassification rate, n (%)
All cases	20 (50.0%)
Stenosis ≥ 50%	10 (58.8%)
Stenosis < 50%	10 (43.5%)

AI-QCPA, artificial intelligence quantified coronary plaque analysis; CCTA, coronary computed tomographic angiography.

The primary reclassification effect was to intensify preventive medical therapy.

## Discussion

In the DECODE study, we have shown that adding AI-QCPA to standard CCTA reports or interpretations may alter medical management recommendations 66% of the time. Of note, there was a high level of medical therapy chosen based on demographic characteristics, risk factors, and CCTA report alone, with 94% of patients recommended for stage 2 medical therapy (Central Illustration). This may be largely attributable to the fact that aspirin therapy and high-intensity statin therapy are justified for most patients with symptoms suggestive of CAD in whom CCTA shows any coronary atherosclerosis. When AI-QCPA information was added, the majority of management changes consisted of up-classification and intensification of medical therapy. We found a high rate of medical therapy reclassification across the spectrum of CCTA-defined CAD, with reclassification rates ranging from 47% in patients with CAC = 0% to 96% in patients with CAC >400, and 89.5% in patients with coronary stenosis >50%. The RR was 89.5% with CAD-RADS scores ≥3 (>50% stenosis), consistent with the CAD-RADS 2.0 document which recommends more aggressive medical therapy with CAD-RADS 3 stenosis or higher (>50% stenosis). That said, 51.6% of patients with <50% stenoses were also reclassified, reflecting a greater appreciation of plaque burden than was reflected in the CCTA report.

Of note, AI-QCPA use did not change the FFR_CT_ ordering pattern in the presence of stenosis ≥50%, but it did significantly alter FFR_CT_ ordering when CCTA showed <50% stenosis. Although FFR_CT_ is recommended for 40% to 90% stenoses, AI-QCPA analysis may give the reader better insight into identification of the type and complexity of plaque, which may help risk-stratify mild stenoses more appropriate for FFR_CT_ analysis.

The focus of the DECODE study was behavior change in clinical management by providers and sought to be generalizable beyond cardiologists with CCTA-interpretation training. For the primary analysis, therefore, the investigators used only the CCTA structured report rather than direct review of the CCTA images. Interestingly, in an exploratory analysis when the investigators, (who were in fact also trained in CCTA interpretation), were shown the CCTA images alongside the structured report for each patient, there was only a modest reduction in the RR (from 66% to 50% reclassification). This observation suggests that whether with anatomic reporting by CAD-RADS 2.0 or direct inspection of the CCTA by trained physicians, there is an underappreciation of the total burden and types of plaque. Thus, the additional information on TPV as well as the CCTA images were incrementally beneficial in management decisions.

There are several reasons that incorporation of AI-QCPA into decision-making may lead to such frequent changes in management, including efforts to align preventive medical therapies with the actual burden of plaque. Insights into patient-specific details of plaque type and quantity are not possible under conventional CCTA reporting paradigms. Although the impact of these factors is well described, there has not previously been the ability to know and therefore act on them. In the Scottish Computed Tomography of the Heart trial, low-attenuation plaque burden was the strongest predictor of myocardial infarction independent of cardiovascular risk score, CAC, or stenosis severity: patients with low-attenuation plaque burden greater than 4% were almost 5 times as likely to have subsequent myocardial infarction.^7^ In the Incident Coronary Syndromes Identified by Computed Tomography case-control sub-study of the Coronary CT Angiography Evaluation for Clinical Outcomes: An International Multicenter registry, 65% of patients who developed acute coronary syndrome had a baseline CCTA showing nonobstructive coronary atherosclerosis, with noncalcified plaque volume and high-risk and necrotic plaque identifying patients at higher risk.^8^^,^^9^ Furthermore, in a secondary analysis of the Prospective Multicenter Imaging Study for Evaluation of Chest Pain cohort, the presence of high-risk plaque including low-attenuation plaque carried a strong association with subsequent cardiovascular events that persisted after adjustment of atherosclerosis cardiovascular disease risk score and stenosis severity.^10^

Recently, age and sex specific nomograms were developed using data from 11,808 patients referred for CCTA who underwent AI-QCPA. These nomograms may prove to be particularly useful in younger patients in whom the absolute plaque burden values may be low, yet may reflect a higher percentile for age and sex. These nomograms may help to identify patients who are on an adverse trajectory and may benefit from aggressive modification of risk with medical therapies.^11^

Given the relatively recent Guideline class IA indication for CCTA for the evaluation of both stable and acute chest pain syndromes, it is expected that this imaging modality will increasingly be utilized by primary care providers.^12^ Much in the way that primary care providers have adopted use of coronary calcium scores to assess cardiovascular risk and guide medical therapy, the quantitative AI description of plaque burden on CCTA is a more accurate assessment of plaque burden and composition than can be ascertained on review of images, let alone a clinical CCTA report. Certain studies have suggested that certain TPV thresholds are associated with higher risk of cardiovascular events.^13^^,^^14^ This information could enable providers to gain a more granular cardiovascular risk assessment and identify more appropriate thresholds for preventive care which are currently being underestimated by risk assessment scores. Further, the numerical quantification of noncalcified and low-attenuation plaque burden could be an attractive way for providers and patients to objectively assess response to therapeutic interventions and potentially identify patients with residual risk factors at an earlier stage in their disease process.

This study is not without limitations. There was variability among readers and institutions in the descriptive elements included within the CCTA reports. For example, the CAD-RADS 2.0 classification was not always stated in reports, which in its newest iteration encompasses presence of high-risk plaque features and plaque burden.^15^ While this may have impacted choice of baseline medication management, the effect would have been mitigated with direct review of CCTA images rather than reports. Another limitation was that decisions regarding medical therapy were not based on cost considerations. In the real world, prior authorizations, requisite insurance approvals, and out-of-pocket costs to the patient may ultimately factor into the timely adoption of certain medical interventions. Lastly, this was a “proof of concept” study identifying theoretical changes in medical decision-making based on review of actual patient data and CCTA reports and images by an expert panel of prevention and imaging-focused cardiologists. These findings should be confirmed in real-world prospective observational data and potentially randomized controlled trials to determine whether such medication management changes are associated with changes in downstream cardiovascular outcomes.

In conclusion, our findings reveal that incorporation of AI-QCPA information into CCTA reporting has the potential to better align treatment strategies with individual patient risk, primarily by intensification of medical therapy.

## References

[1] Khasanova E., Indraratna P., Miranda P. (2022). Head to head comparison reproducibility and inter-reader agreement of an AI based coronary stenosis algorithm vs level 3 readers. J Cardiovasc Comput Tomogr.

[2] Narula J., Stuckey T., Nakazawa G. (2023). Primary results of the REVEALPLAQUE study: A prospective quantitative assessment of AI-based CCTA plaque volume compared with IVUS. JCCT.

[3] Petersen K., Schaap M., Mirza S. (2022). Quantitative assessment of AI-based CCTA plaque volume compared with IVUS. J Cardiovasc Comput Tomogr.

[4] Dundas, et al. Interaction of AI-enabled quantitative coronary plaque volumes on coronary CT angiography, FFRCT, and clinical outcomes: a retrospective analysis of the ADVANCE registry. Circ Cardiovasc Imaging. In press. 10.1161/CIRCIMAGING.123.016143 38469689

[5] Freeman A.M., Raman S.V., Aggarwal M. (2023). Integrating coronary atherosclerosis burden and progression with coronary artery disease risk factors to guide therapeutic decision making. Am J Med.

[6] Douglas P.S., Nanna M.G., Kelsey M.D. (2023). Comparison of an initial risk-based testing strategy vs usual testing in stable symptomatic patients with suspected coronary artery disease: the PRECISE randomized clinical trial. JAMA Cardiol.

[7] Williams M.C., Kwiecinski J., Doris M. (2020). Low-attenuation noncalcified plaque on coronary computed tomography angiography predicts myocardial infarction: results from the multicenter SCOT-HEART trial (Scottish Computed Tomography of the HEART). Circulation.

[8] Chang H.J., Lin F.Y., Lee S.E. (2018). Coronary atherosclerotic precursors of acute coronary syndromes. J Am Coll Cardiol.

[9] Ferraro R.A., van Rosendael A.R., Lu Y. (2020). Non-obstructive high-risk plaques increase the risk of future culprit lesions comparable to obstructive plaques without high-risk features: the ICONIC study. Eur Heart J Cardiovasc Imaging.

[10] Ferencik M., Mayrhofer T., Bittner D.O. (2018). Use of high-risk coronary atherosclerotic plaque detection for risk stratification of patients with stable chest pain: a secondary analysis of the PROMISE randomized clinical trial. JAMA Cardiol.

[11] Tzimas G., Gulsin G.S., Everett R.J. (2023). Age- and sex-specific nomographic CT quantitative plaque data from a large international cohort. JACC Cardiovasc Imaging.

[12] Gulati M., Levy P.D., Writing Committee Members (2021). 2021 AHA/ACC/ASE/CHEST/SAEM/SCCT/SCMR Guideline for the Evaluation and Diagnosis of Chest Pain: Executive Summary: a report of the American College of Cardiology/American Heart Association Joint Committee on Clinical Practice Guidelines. J Am Coll Cardiol.

[13] Deseive S., Kupke M., Straub R. (2021). Quantified coronary total plaque volume from computed tomography angiography provides superior 10-year risk stratification. Eur Heart J Cardiovasc Imaging.

[14] Lin A., Manral N., McElhinney P. (2022). Deep learning-enabled coronary CT angiography for plaque and stenosis quantification and cardiac risk prediction: an international multicentre study. Lancet Digit Health.

[15] Cury R.C., Leipsic J., Abbara S. (2022). CAD-RADS™ 2.0-2022 Coronary Artery Disease-Reporting and Data System: an expert consensus document of the Society of Cardiovascular Computed Tomography (SCCT), the American College of Cardiology (ACC), the American College of Radiology (ACR), and the North America Society of Cardiovascular Imaging (NASCI). JACC Cardiovasc Imaging.

